# Glutamine and glutamate limit the shortening of action potential duration in anoxia-challenged rabbit hearts

**DOI:** 10.14814/phy2.12535

**Published:** 2015-09-02

**Authors:** Kenneth J Drake, Matthew S Shotwell, John P Wikswo, Veniamin Y Sidorov

**Affiliations:** 1 Department of Molecular Physiology and Biophysics, Vanderbilt UniversityNashville, Tennessee; 2 Vanderbilt Institute for Integrative Biosystems Research and Education, Vanderbilt UniversityNashville, Tennessee; 3 Department of Biostatistics, Vanderbilt UniversityNashville, Tennessee; 4 Department of Physics and Astronomy, Vanderbilt UniversityNashville, Tennessee; 5 Department of Biomedical Engineering, Vanderbilt UniversityNashville, Tennessee

**Keywords:** Action potential duration, amino acids, anoxia

## Abstract

In clinical conditions, amino acid supplementation is applied to improve contractile function, minimize ischemia/reperfusion injury, and facilitate postoperative recovery. It has been shown that glutamine enhances myocardial ATP/APD (action potential duration) and glutathione/oxidized glutathione ratios, and can increase hexosamine biosynthesis pathway flux, which is believed to play a role in cardioprotection. Here, we studied the effect of glutamine and glutamate on electrical activity in Langendorff-perfused rabbit hearts. The hearts were supplied by Tyrode's media with or without 2.5 mmol/L glutamine and 150 *μ*mol/L glutamate, and exposed to two 6-min anoxias with 20-min recovery in between. Change in APD was detected using a monophasic action potential probe. A nonlinear mixed-effects regression technique was used to evaluate the effect of amino acids on APD over the experiment. Typically, the dynamic of APD change encompasses three phases: short transient increase (more prominent in the first episode), slow decrease, and fast increase (starting with the beginning of recovery). The effect of both anoxic challenge and glutamine/glutamate was cumulative, being more pronounced in the second anoxia. The amino acids' protective effect became largest by the end of anoxia – 20.0% (18.9, 95% CI: [2.6 ms, 35.1 ms]), during the first anoxia and 36.6% (27.1, 95% CI: [7.7 ms, 46.6 ms]), during the second. Following the second anoxia, APD difference between control and supplemented hearts progressively increased, attaining 10.8% (13.6, 95% CI: [4.1 ms, 23.1 ms]) at the experiments' end. Our data reveal APD stabilizing and suggest an antiarrhythmic capacity of amino acid supplementation in anoxic/ischemic conditions.

## Introduction

Cardiovascular disease is a leading cause of mortality in the United States, accounting for 33.6% of all deaths (Roger et al. 2011). The heart's high metabolic demands and high work output make it highly susceptible to damage from any disruption in the supply of either oxygen or metabolic substrates (Taegtmeyer 1994). In many types of heart disease and dysfunction, metabolism is the first area affected, which can then lead to different deleterious consequences, such as ion imbalance, decreased contractile function, increased free radical production, cardiomyopathy, arrhythmias, and sudden cardiac death (Guertl et al. 2000; Hutcheson and Rocic 2012). To decrease the impact of substrate deprivation, the heart is quite flexible in its ability to metabolize many different substrates, including fatty acids, glucose, lactate, ketone bodies, and amino acids. Because oxidative phosphorylation plays a central role in the generation of ATP, a decrease in oxygen supply immediately leads to alterations in heart metabolism and affects cardiac pump efficiency.

Cardiac hypoxia/anoxia takes place during heart failure, carbon monoxide poisoning, drowning and other events where oxygen supply is interrupted without blockage of coronary arteries. While cardiac hypoxia/anoxia may not be as common or clinically applicable as cardiac ischemia, it allows dissection, and study of the physiological effects particularly related to oxygen deprivation without the sequelae associated with complete ischemia. During hypoxia/anoxia the heart receives adequate substrate throughput, and the blockage of oxidative phosphorylation can be isolated from the effects of waste accumulation or substrate depletion, as occur with no-flow ischemia.

Glucose, fatty acids, and other substrates require oxygen for full energy yield and produce significant levels of acidic by-products (Taegtmeyer et al. 1997). Amino acids are of particular interest due to their potential for nonoxidative metabolism and their low contribution to cellular acidification. In addition to their role as protein precursors, amino acids are essential components in many aspects of physiology and can serve as metabolic substrates (Drake et al. 2012). We hypothesized that supplementation with glutamate and glutamine maintains electrical properties of the heart during anoxia and improves electrical stability in the recovery period.

Glutamine and glutamate were chosen because glutamate can be easily transaminated to *α*-ketoglutarate and metabolized via the TCA cycle (Cohen et al. 2003). It is also a one-step transamidation reaction to turn glutamine into glutamate, making the two amino acids highly interconvertible in the cell (Stottrup et al. 2006). Once the amino acids enter the TCA cycle as *α*-ketoglutarate, they can be converted to succinate, yielding one molecule each of GTP and NADH (Julia et al. 1990). This anaplerotic reaction also serves to maintain the levels of TCA cycle intermediates so that the metabolic machinery is primed to resume normal function once oxygen returns.

The metabolic and electrophysiological response to ischemia can deviate depending on multiple factors, such as vasculature, hormonal status, heart rate and metabolic rate, concomitant diseases, gender, and age (Freedberg et al. 1970; Ostadal et al. 1984; Hearse 1998; Fazio et al. 2004). The same factors could also underlie interspecies variability in the case of anoxia, and thereby complicate analysis of the response to anoxia, especially when anoxic stress is of short or moderate duration.

To account for the variability among hearts in the effects of anoxia on action potential duration (APD) and to quantify statistical uncertainty in our work, we utilized nonlinear, mixed-effects regression (Pinheiro and Bates 2000). Our findings demonstrate the importance of amino acids in the maintenance of electrophysiological properties of the heart in conditions of loss of oxidative phosphorylation, and suggest a cardioprotective and antiarrhythmic potential of amino acid supplementation in response to transient anoxia/ischemia with few apparent side effects.

## Methods

All experiments conformed to the *Guide for the Care and Use of Laboratory Animals* published by the U.S. National Institutes of Health and were approved in advance by the Vanderbilt Institutional Animal Care and Use Committee.

### Experimental preparation

In our experiments, New Zealand white rabbits of either gender (16 males and 11 females, *N *=* *27) weighing 2.7–3.1 kg were used. In the current work, we did not seek to explore gender differences in response to anoxia, although certain sex-dependent disparities exist in both cardiac ischemic injury (Johnson et al. 2006) and postischemic recovery (Mendelsohn and Karas 2005; Besik et al. 2007), which could affect data distribution. We believe that our statistical approach for data analysis can reliably detect the effect of amino acids supplementation regardless of gender-related data variability. The detailed description of the heart preparation has been published previously (Sidorov et al. 2007, 2008). The animals were preanesthetized with ketamine (50 mg/kg), heparinized (1000 units), and anesthetized with sodium pentobarbital (60 mg/kg). After midsternal incision, the hearts were quickly removed from the chest and mounted on a Langendorff apparatus for retrograde perfusion with oxygenated Tyrode's solution of the following composition (in mmol/L): 133 NaCl, 4 KCl, 2 CaCl_2_, 1 MgCl_2_, 1.5 NaH_2_PO_4_, 20 NaHCO_3_ and 10 d-glucose, buffered with 95% O_2_ and 5% CO_2_ (pH = 7.35 at 37°C). The coronary perfusion pressure was adjusted to 50 mmHg. The experimental setup was located in a Faraday shield, and the temperature inside was kept at 37 ± 0.5°C by a precision heater controller (Air-Therm, World Precision Instruments, Sarasota, FL).

Hearts assigned to the experimental group received the Tyrode's solution supplemented with 2.5 mmol/L glutamine and 150 *μ*mol/L glutamate. While glutamine is the primary focus of this study, glutamate was also included in the media to reduce washout of the cellular stores (Suleiman et al. 1997a,b), since anoxia leads to ATP depletion (Allen and Orchard 1987; Taegtmeyer 1994) and inhibits the function of the active transport system that maintains ion balance essential to support glutamate influx (Dinkelborg et al. 1995). The glutamine level is much higher than its normal concentration (390–650 *μ*mol/L), whereas the glutamate level is only slightly higher than its normal concentration in the blood from healthy adults (18–98 *μ*mol/L) (A.D.A.M., 2013). During baseline, before anoxia was applied, the average APD across control and experimental groups was 153.5 msec (95% CI: [149.2 ms, 157.7 ms]).

### Stimulation and registration protocol

Stimulation was continuously maintained at a cycle length of 300 msec delivered via a bipolar glass-coated platinum electrode (0.25-mm wire diameter; 1-mm electrode separation) inserted into the cavity, close to the apex of the left ventricle. The stimulus was 4 msec in duration, with strength adjusted to between three and four times of the threshold. A monophasic action potential (MAP) was recorded using a commercially available standard MAP catheter (1675P; EP Technologies, Sunnyvale, CA). While MAP is an extracellularly recorded wave form, it closely reproduces the course of the repolarization phase of the transmembrane action potential and has been broadly utilized in both clinical and experimental conditions to obtain stable recordings to monitor APD (Franz 1991, 1999; Banville and Gray 2002; Knollmann et al. 2007). The MAP probe was positioned on the epicardium close to the septum on the anterior side of the heart and held perpendicular to the surface. The signals were amplified with a differential amplifier (DP-301; Warner Instruments, LLC, Hamden, CT) and then visualized and recorded at 25 kHz with a digital oscilloscope (TDS5034B; Tektronix, Beaverton, OR).

### Experimental protocol

In our previous experiments, we found that 4 min of anoxia resulted in rapid recovery and no long-term effect on APD, whereas 8 min and longer resulted in increasing incidences of arrhythmias and increasingly severe APD depression. Therefore, 6 min of anoxia were chosen to have a substantial decrease in APD without inducing fibrillation or a loss of pacing. The interval of 20 min was used as an adequate time for re-equilibration after each anoxic episode.

After an equilibration time for 30 min, baseline MAPs were recorded every 4 min for 20 min. The heart was then switched from oxygen to nitrogen-saturated media. The recordings were taken every minute during the 6-min anoxic episode. The perfusion was then switched back to oxygenated media for the recovery period of 20 min. During this time recordings were taken every minute for 6 min, then every 2 min for the next 6 min. The last two recordings were done at 15 and 20 min postanoxia. Thereafter, the anoxia and recovery cycle was repeated.

### Data processing and statistical analysis

The MAP data were analyzed using MATLAB software (MathWorks, Natick, MA). The acquired data were postprocessed using a 5 kHz digital low pass filter. The activation time was computed based on (*dV*/*dt*)_max_ criteria and repolarization time was measured at 90% repolarization. APD was computed as a time difference between activation and repolarization times. Each value of APD was computed as the average of 6–8 sequential APDs.

Action potential duration was modeled as follows: APD(*t*) = *k*_0_ + *k*_1_[*x*_0_(*t*) − *x*_1_(*t*)] − *k*_2_*t*^2^, where *k*_0_ is the baseline APD, and *x*_0_(*t*) and *x*_1_(*t*) are solutions to a pair of piecewise linear differential equations taking the form 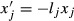 during anoxia, and 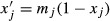 otherwise, for *j*=1, 2 and *x*_0_(0) = *x*_1_(0) = 1. Thus, the effect of anoxia on APD was modeled as an exponential decay, followed by exponential recovery upon reoxygenation. Although 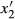 enters the anoxia state immediately, 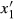 remains in the baseline state for a period of time determined by an offset parameter. This offsetting captures the transient elongation of APD just after the start of anoxia. The last term, *k*_2_*t*^2^, models the secular trend (decay) in APD over the entire experiment (Shotwell et al. 2013). Nonlinear mixed-effects regression (Pinheiro and Bates 2000) was used to account for the variability among hearts (random effects), and to quantify the effects of amino acid supplementation (fixed effects) on each of the model parameters and the resulting APD in response to anoxia challenges. The mixed-effects method is similar to a “two-stage approach,” in which the statistical model is fitted separately for each heart (stage 1), and the resulting model fits or their summaries are compared across treatment groups (stage 2). While simple, this approach fails to “propagate” statistical uncertainty to the second stage, and cannot be used when only partial data are available for some hearts. The mixed-effects method combines the steps of the two-stage approach for a parsimonious solution. The differences in the average APD between groups over the course of the experiment were summarized using 95% confidence intervals (confidence band). Intervals that exclude the appropriate null value (zero) were considered statistically significant.

## Results

**Figure 1:**
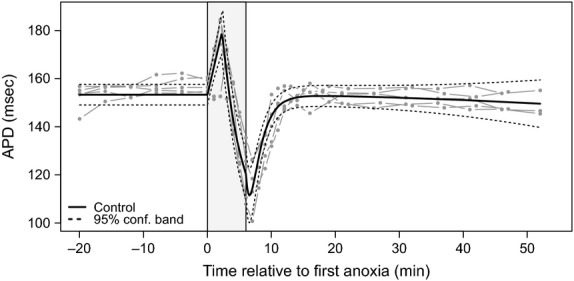
Action potential duration (APD) stability during long recovery phase after single anoxia. For each experimental replicate (*N *=* *5), the APD time series is illustrated in light gray. For the purpose of graphical presentation, each original time series was adjusted to the overall preanoxia mean. The solid black line represents mean APD from the fitted model. The dotted black lines define a pointwise 95% confidence band. The vertical gray bar delineates the period of anoxia challenge.

To assess (1) if amino acid supplementation affects APD under normal conditions and (2) how much time is required for APD to recover after anoxia, a series of preliminary experiments were conducted. In one set of experiments (*N *=* *6), the heart was initially perfused with regular Tyrode's solution for 20 min, and then the perfusion system was switched to supply the heart with enriched Tyrode's solution for the next 20 min. The addition of amino acids insignificantly increased APD from 150.5 ms (95% CI: [148.5 ms, 152.4 ms]) to 153 ms (95% CI: [149.9 ms, 156.1 ms]) (*N *=* *24) (six experiments, unpaired *t*-test). Another set of experiments (*N *=* *5), wherein the hearts were exposed only to one anoxia, is presented in Figure 1. As the figure illustrates, mean APD recovers to 99% (152.7 ms, 95% CI: [148.3 ms, 157.0 ms]) of baseline APD within 10 min following cessation of anoxia; therefore, 20 min was chosen as the sufficient recovery interval between two sequential anoxias.

**Figure 2:**
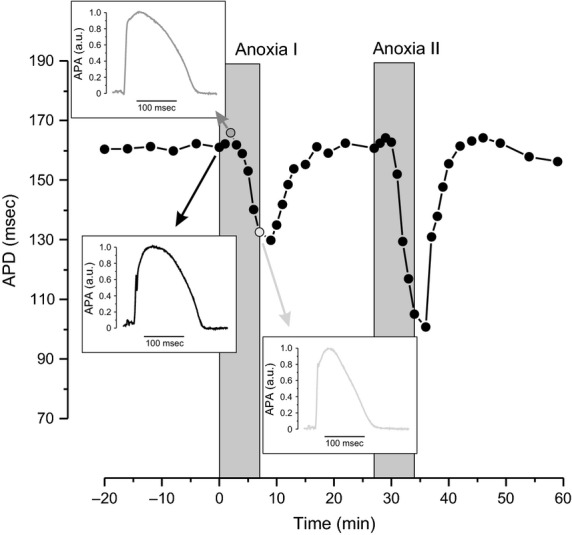
The typical double anoxia experiment when successive 6-min anoxia is followed by 20 min of recovery. The insets demonstrate monophasic action potential recorded at different phases of anoxia-induced action potential duration (APD) change.

Figure 2 demonstrates the characteristic data of APD change in response to two successive anoxias. In this control experiment, the heart was perfused with only glucose-containing media. Three phases can be readily distinguished in each set of anoxia. The first phase of abrupt APD increase starts immediately after onset of anoxia. This initial change in APD was more pronounced in the first insult of anoxia and was observed in 18 of 21 conducted experiments. The first transient phase is followed by considerable APD decrease, which lasts until cessation of anoxia. This APD shortening was always greater during the second period of anoxia. The third phase of anoxia-induced change in APD takes place after recommencing the perfusion with oxygenated solution. The fast APD recovery phase continues for about 10 min. The insets in Figure 2 illustrate MAP traces recorded at different phases of APD change. The described APD dynamics were typical in both control and hearts perfused with enriched media.

**Figure 3:**
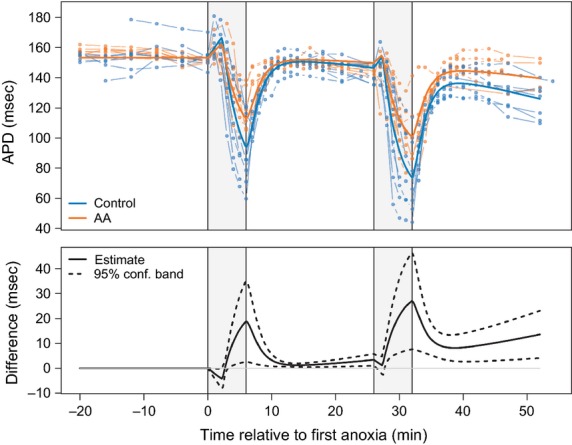
The dynamics of action potential duration (APD) change in response to two sequential insults of anoxia. Vertical gray bars show the two periods of anoxia. Upper panel: The solid blue and red colored lines depict mean APD for control (*N *=* *8) and amino acids supplemented (*N *=* *8) hearts, respectively. The dot–dash colored lines represent individual APD time series for each replicate. For the purpose of graphical presentation, each original time series was adjusted to the overall preanoxia mean. Lower panel: Estimated difference in mean APD for enriched versus normally perfused hearts with pointwise 95% confidence band.

Figure 3 represents the effect of glutamine/glutamate supplementation on mean APD dynamics in response to two insults of anoxia (*N *=* *16). The upper panel in Figure 3 illustrates estimated mean APD curves from control (blue) and from experimental (orange) hearts with individual time series data superimposed in lighter color. The lower panel shows the estimated difference in mean APD between control and enriched groups and the 95% confidence band. After onset of the first anoxia, the first phase of acute transient APD increase peaks at 2.27 min (95% CI: [2.01 min, 2.54 min]) with amplitude of 13.7 ms (95% CI: [11.8 ms, 15.7 ms]) and 8.1 ms (95% CI: [9.4 ms, 10.7 ms]) above baseline for control and supplemented media groups, respectively. The subsequent APD shortening is 39% (59.3 ms, 95% CI: [51.0 ms, 67.7 ms]) for control and 26% (40.5 ms, 95% CI: [32.4 ms, 48.6 ms]) for experimental groups. In the course of the second anoxia, the first phase transient APD elongation peaks at 1.3 min (95% CI: [1.0 min, 1.7 min]), which is about 0.9 min (95% CI: [0.5 min, 1.3 min]) faster than in the first anoxia. The magnitude (relative to mean APD just before the start of anoxia) is 5.1 ms (95% CI: [−0.4 ms, 10.8 ms]) and 7.4 ms (95% CI: [−2.0 ms, 16.7 ms]) for supplemented and normal hearts, respectively. The following APD shortening is 48% (73.7 msec, 95% CI: [59.0 msec, 88.4 msec]) in the hearts perfused with regular solution and 32.0% (100.8 msec, 95% CI: [86.2 msec, 115.2 msec]) in the hearts supplied with amino acids.

Thus, the control group shows a significantly larger drop in APD than the supplemented group. The difference in dynamics of APD change in response to amino acid administration can be easily detected from the estimated difference in mean APD for enriched versus normally perfused hearts, as illustrated in the lower panel of Figure 3. It should be noted that anoxia induces gradual APD depression over the entire experiment. The rate of APD decay over the course of the experiments in the amino acid supplemented hearts (0.010 ms/s/s, 95% CI: [0.005, 0.015]) was less than in control (0.020 ms/s/s, 95% CI: [0.015, 0.025]). Notably, there was no evidence of significant decay in APD among hearts exposed to a single anoxia event (0.003 ms/s/s, 95% CI: [0.003, 0.009]). On average, the APDs for control hearts were 10.8% shorter (13.6 msec, 95% CI: [4.1 msec, 23.1 msec]) than hearts supplemented with glutamine and glutamate at the end of the experiment.

## Discussion

We investigated the effects of amino acid supplementation on the dynamics of APD in response to two consecutive insults of anoxia. It is known that glutamate is at high tissue–plasma ratios in the heart and is associated with increased contractile strength, NADH/NAD^+^ ratios, and ATP levels when given to hearts before anoxia (Dinkelborg et al. 1996). Glutamine supplementation has also been shown to improve contractile function and Ca^2+^ homeostasis during chemical hypoxia (Suleiman et al. 1997a; Williams et al. 2001). Increased intracellular glutamate stores are correlated with improved metabolic recovery, increased cardiac output, and elevated glutathione levels after an ischemic episode (Wischmeyer et al. 2003; Stottrup et al. 2006; Bolotin et al. 2007). However, glutamate is a potent neurotransmitter and elevated levels of circulating glutamate have been associated with neurological side effects (Graham et al. 2000; Wischmeyer et al. 2003). Glutamine, on the other hand, has no known toxicity threshold and is normally the amino acid of highest concentration in circulation (Lomivorotov et al. 2011). A single dose of oral or intravenous glutamine has been shown to improve tissue glutamate levels without the need for high levels of glutamate in the plasma (Engel et al. 2009; Todorova et al. 2009; Cheng et al. 2012; Weitzel et al. 2013). This has important clinical applications, because if it can be shown that glutamine improves disease outcomes, there would be a wide therapeutic range and negligible risks associated with its use.

At onset of anoxia a rapid and substantial increase in APD occurs. Such behavior was observed in 18 of 21 investigated hearts within 3 min of anoxia and was independent of amino acid supplementation. This phenomenon of initial prolongation of APD after metabolic inhibition has been described previously by Carmeliet and Boulpaep (1957) in the frog heart and later in sheep Purkinje fibers (Carmeliet 1978). Isenberg et al. (1983) suggested that initial prolongation of APD in guinea-pig ventricular myocytes results from block of the electrogenic Na pump due to ATP depletion. The biphasic response in rabbit and human cardiomyocytes to hypoxia and metabolic inhibition has been reported by Verkerk et al. (1996). They detected this phenomenon in all subepicardial myocytes, but not in most subendocardial myocytes. They found that a decrease in *I*_to_, which occurs prior to the opening of K_ATP_ channels, underlies the metabolic-induced initial increase in APD.

In our work, the application of amino acids significantly decreased anoxia-induced APD shortening by 29.9% (18.9 ms, 95% CI: [2.6 ms, 35.1 ms]) in the first anoxia and by 36.6% (27.1 ms, 95% CI: [7.7ms, 46.6 ms]) in the second period of anoxia. In addition, the amino acids significantly reduced slow APD decay. The observed cardioprotective effect of amino acids can be mediated via limiting the damage incurred during anoxia/reoxygenation. It has been shown that glutamine can enhance synthesis of intracellular glutathione (Hong et al. 1992) and increase the reduced-oxidized glutathione ratio (Khogali et al. 2002) and effect on redox state of the cells (Engel et al. 2009).

Glutamine is also required for metabolism of glucose into the hexosamine biosynthesis pathway (HBP) to be used for glycosylation of nuclear and cytoplasmic proteins (Lauzier et al. 2013). Increasing glutamine concentration results in significant elevation of uridine diphosphate *N*-acetyl-glucosamine, which is the obligatory substrate for the transfer of a single *N*-acetyl-glucosamine (O-GlcNAc) to proteins by O-GlcNAc transferase (Love and Hanover 2005). Alteration in glycosylation can have a direct effect on multiple phosphorylation cascades (Filippis et al. 1997; Singh and Crook 2000; Burt et al. 2003) and be involved in response to anoxic insult. It has been shown in isolated cardiomyocytes that an increased O-GlcNAc level reduces the ischemia–reperfusion-induced apoptosis (Champattanachai et al. 2007) and attenuates the loss of mitochondrial membrane potential due to oxidative stress (Champattanachai et al. 2008). The dynamic addition of O-GlcNAc to proteins can prevent protein degradation (Zachara and Hart 2006) and lead to reduction of ischemia–reperfusion-generated calcium influx (Champattanachai et al. 2007).

Another possible mechanism for amino acid-mediated metabolic support is anaplerosis, when replacement of depleted intermediates takes place. Glutamate and glutamine are interconvertible via transamination reaction (Ottaway 1969; Stottrup et al. 2006; Lofgren et al. 2010), and then glutamate can be transaminated to enter the TCA cycle as *α*-ketoglutarate (Cohen et al. 2003). The additional supply of intermediates could support the return of oxidative phosphorylation during reoxygenation. However, Lauzier et al. recently demonstrated that glutamine has a marginal anaplerotic potential, regardless of substrate availability, but it significantly enhances the contribution of exogenous long chain fatty acids to *β*-oxidation and triglyceride formation. These metabolic effects of glutamine were reversed by azaserine, a structural analog of glutamine, which inhibits glucose entry into the HBP. The authors suggest the recuperative role of the glutamine as HBP-mediated glycosylation of the fatty acid transporter CD36 (Lauzier et al. 2013). In our experiments, in addition to glutamine/glutamate, only glucose was utilized as a substrate for energy metabolism; therefore, glycosylation of the fatty acid transporter CD36 could not play a role in amino acids' protective effect.

It should be noted that though the model of the regional anoxia in our work would be more clinically relevant than global anoxia, the anoxia/ischemia in regional model creates very heterogeneous metabolic and electrophysiological changes, which would significantly impede and complicate already complex data analysis. Conversely, the global model minimizes the spatial gradient of metabolic and electrophysiological properties and thereby allows dissecting the effect of amino acids on APD. We are confident that the effect of glutamine/glutamate supplementation on the dynamics of the anoxia–APD relationship is fully pertinent to cardiac tissue suffering from local anoxia/ischemia.

## Conclusions

In this work, we investigated, for the first time to the best of our knowledge, the effect of amino acid administration on the electrical properties of the heart during two transitional periods of anoxia. Because of the variability among hearts in baseline APD, and in their response to anoxic events, conventional statistical analysis (e.g., using a series of two-sample *t*-tests) was not appropriate. We accounted for this variability by fitting a simple mechanistic model using nonlinear mixed-effects regression. Thus, we could clearly distinguish the effect of amino acid supplementation on mean APD dynamics in response to anoxia challenge. Our findings provide valuable insights into the interplay of metabolism and electrophysiology by showing that mitigation of energy deficits has immediate effects on electrical function, which may improve survival rates during anoxic or ischemic events.

## References

[b1] A.D.A.M Atlanta, GA A.D.A.M. Inc (2013). http://www.nlm.nih.gov/medlineplus/ency/article/003361.htm.

[b2] Allen DG, Orchard CH (1987). Myocardial contractile function during ischemia and hypoxia. Circ. Res.

[b3] Banville I, Gray RA (2002). Effect of action potential duration and conduction velocity restitution and their spatial dispersion on alternans and the stability of arrhythmias. J. Cardiovasc. Electrophysiol.

[b4] Besik J, Szarszoi O, Kunes J, Netuka I, Maly J, Kolar F (2007). Tolerance to acute ischemia in adult male and female spontaneously hypertensive rats. Physiol. Res.

[b5] Bolotin G, Raman J, Williams U, Bacha E, Kocherginsky M, Jeevanandam V (2007). Glutamine improves myocardial function following ischemia–reperfusion injury. Asian Cardiovasc. Thorac. Ann.

[b6] Burt DJ, Gruden G, Thomas SM, Tutt P, Dell'Anna C, Viberti GC (2003). P38 mitogen-activated protein kinase mediates hexosamine-induced TGFbeta1 mRNA expression in human mesangial cells. Diabetologia.

[b7] Carmeliet E (1978). Cardiac transmembrane potentials and metabolism. Circ. Res.

[b8] Carmeliet E, Boulpaep E (1957). Effect of 2,4-dinitrophenol on the duration of action potential of the ventricular muscle in frog. C. R. Seances Soc. Biol. Fil.

[b9] Champattanachai V, Marchase RB, Chatham JC (2007). Glucosamine protects neonatal cardiomyocytes from ischemia–reperfusion injury via increased protein-associated O-GlcNAc. Am. J. Physiol. Cell Physiol.

[b10] Champattanachai V, Marchase RB, Chatham JC (2008). Glucosamine protects neonatal cardiomyocytes from ischemia–reperfusion injury via increased protein O-GlcNAc and increased mitochondrial Bcl-2. Am. J. Physiol. Cell Physiol.

[b11] Cheng S, Rhee EP, Larson MG, Lewis GD, McCabe EL, Shen D (2012). Metabolite profiling identifies pathways associated with metabolic risk in humans. Circulation.

[b12] Cohen DM, Guthrie PH, Gao X, Sakai R, Taegtmeyer H (2003). Glutamine cycling in isolated working rat heart. Am. J. Physiol. Endocrinol. Metab.

[b13] Dinkelborg LM, Kinne RK, Grieshaber MK (1995). Characterization and pH dependence of l-glutamate transport in sarcolemmal vesicles from rat hearts. Am. J. Physiol.

[b14] Dinkelborg LM, Kinne RK, Grieshaber MK (1996). Transport and metabolism of l-glutamate during oxygenation, anoxia, and reoxygenation of rat cardiac myocytes. Am. J. Physiol.

[b15] Drake KJ, Sidorov VY, McGuinness OP, Wasserman DH, Wikswo JP (2012). Amino acids as metabolic substrates during cardiac ischemia. Exp. Biol. Med. (Maywood).

[b16] Engel JM, Muhling J, Kwapisz M, Heidt M (2009). Glutamine administration in patients undergoing cardiac surgery and the influence on blood glutathione levels. Acta Anaesthesiol. Scand.

[b17] Fazio S, Palmieri EA, Lombardi G, Biondi B (2004). Effects of thyroid hormone on the cardiovascular system. Recent Prog. Horm. Res.

[b18] Filippis A, Clark S, Proietto J (1997). Increased flux through the hexosamine biosynthesis pathway inhibits glucose transport acutely by activation of protein kinase C. Biochem. J.

[b19] Franz MR (1991). Method and theory of monophasic action potential recording. Prog. Cardiovasc. Dis.

[b20] Franz MR (1999). Current status of monophasic action potential recording: theories, measurements and interpretations. Cardiovasc. Res.

[b21] Freedberg AS, Papp JG, Williams EM (1970). The effect of altered thyroid state on atrial intracellular potentials. J. Physiol.

[b22] Graham TE, Sgro V, Friars D, Gibala MJ (2000). Glutamate ingestion: the plasma and muscle free amino acid pools of resting humans. Am. J. Physiol. Endocrinol. Metab.

[b23] Guertl B, Noehammer C, Hoefler G (2000). Metabolic cardiomyopathies. Int. J. Exp. Pathol.

[b24] Hearse DJ (1998). Myocardial protection during ischemia and reperfusion. Mol. Cell. Biochem.

[b25] Hong RW, Rounds JD, Helton WS, Robinson MK, Wilmore DW (1992). Glutamine preserves liver glutathione after lethal hepatic injury. Ann. Surg.

[b26] Hutcheson R, Rocic P (2012). The metabolic syndrome, oxidative stress, environment, and cardiovascular disease: the great exploration. Exp. Diabetes Res.

[b27] Isenberg G, Vereecke J, van der Heyden G, Carmeliet E (1983). The shortening of the action potential by DNP in guinea-pig ventricular myocytes is mediated by an increase of a time-independent K conductance. Pflugers Arch.

[b28] Johnson MS, Moore RL, Brown DA (2006). Sex differences in myocardial infarct size are abolished by sarcolemmal KATP channel blockade in rat. Am. J. Physiol. Heart Circ. Physiol.

[b29] Julia P, Young HH, Buckberg GD, Kofsky ER, Bugyi HI (1990). Studies of myocardial protection in the immature heart. II. Evidence for importance of amino acid metabolism in tolerance to ischemia. J. Thorac. Cardiovasc. Surg.

[b30] Khogali SE, Pringle SD, Weryk BV, Rennie MJ (2002). Is glutamine beneficial in ischemic heart disease?. Nutrition.

[b31] Knollmann BC, Schober T, Petersen AO, Sirenko SG, Franz MR (2007). Action potential characterization in intact mouse heart: steady-state cycle length dependence and electrical restitution. Am. J. Physiol. Heart Circ. Physiol.

[b32] Lauzier B, Vaillant F, Merlen C, Gelinas R, Bouchard B, Rivard ME (2013). Metabolic effects of glutamine on the heart: anaplerosis versus the hexosamine biosynthetic pathway. J. Mol. Cell. Cardiol.

[b33] Lofgren B, Povlsen JA, Rasmussen LE, Stottrup NB, Solskov L, Krarup PM (2010). Amino acid transamination is crucial for ischaemic cardioprotection in normal and preconditioned isolated rat hearts – focus on l-glutamate. Exp. Physiol.

[b34] Lomivorotov VV, Efremov SM, Shmirev VA, Ponomarev DN, Lomivorotov VN, Karaskov AM (2011). Glutamine is cardioprotective in patients with ischemic heart disease following cardiopulmonary bypass. Heart Surg. Forum.

[b35] Love DC, Hanover JA (2005). The hexosamine signaling pathway: deciphering the “O-GlcNAc code”. Sci. STKE.

[b36] Mendelsohn ME, Karas RH (2005). Molecular and cellular basis of cardiovascular gender differences. Science.

[b37] Ostadal B, Prochazka J, Pelouch V, Urbanova D, Widimsky J (1984). Comparison of cardiopulmonary responses of male and female rats to intermittent high altitude hypoxia. Physiol. Bohemoslov.

[b38] Ottaway JH (1969). Glutamine metabolism in the perfused rat heart. Q. J. Exp. Physiol. Cogn. Med. Sci.

[b39] Pinheiro JC, Bates DM (2000). Mixed-effects models in S and S-PLUS.

[b40] Roger VL, Go AS, Lloyd-Jones DM, Adams RJ, Berry JD, Brown TM (2011). Heart disease and stroke statistics – 2011 update: a report from the American Heart Association. Circulation.

[b41] Shotwell MS, Drake KJ, Sidorov VY, Wikswo JP (2013). Mechanistic analysis of challenge-response experiments. Biometrics.

[b42] Sidorov VY, Woods MC, Baudenbacher F (2007). Cathodal stimulation in the recovery phase of a propagating planar wave in the rabbit heart reveals four stimulation mechanisms. J. Physiol.

[b43] Sidorov VY, Holcomb MR, Woods MC, Gray RA, Wikswo JP (2008). Effects of unipolar stimulation on voltage and calcium distributions in the isolated rabbit heart. Basic Res. Cardiol.

[b44] Singh LP, Crook ED (2000). Hexosamine regulation of glucose-mediated laminin synthesis in mesangial cells involves protein kinases A and C. Am. J. Physiol. Renal. Physiol.

[b45] Stottrup NB, Kristiansen SB, Lofgren B, Hansen BF, Kimose HH, Botker HE (2006). l-glutamate and glutamine improve haemodynamic function and restore myocardial glycogen content during postischaemic reperfusion: a radioactive tracer study in the rat isolated heart. Clin. Exp. Pharmacol. Physiol.

[b46] Suleiman MS, Dihmis WC, Caputo M, Angelini GD, Bryan AJ (1997a). Changes in myocardial concentration of glutamate and aspartate during coronary artery surgery. Am. J. Physiol.

[b47] Suleiman MS, Moffatt AC, Dihmis WC, Caputo M, Hutter J A, Angelini GD (1997b). Effect of ischaemia and reperfusion on the intracellular concentration of taurine and glutamine in the hearts of patients undergoing coronary artery surgery. Biochim. Biophys. Acta.

[b48] Taegtmeyer H (1994). Energy metabolism of the heart: from basic concepts to clinical applications. Curr. Probl. Cardiol.

[b49] Taegtmeyer H, Goodwin GW, Doenst T, Frazier OH (1997). Substrate metabolism as a determinant for postischemic functional recovery of the heart. Am. J. Cardiol.

[b50] Todorova V, Vanderpool D, Blossom S, Nwokedi E, Hennings L, Mrak R (2009). Oral glutamine protects against cyclophosphamide-induced cardiotoxicity in experimental rats through increase of cardiac glutathione. Nutrition.

[b51] Verkerk AO, Veldkamp MW, van Ginneken AC, Bouman LN (1996). Biphasic response of action potential duration to metabolic inhibition in rabbit and human ventricular myocytes: role of transient outward current and ATP-regulated potassium current. J. Mol. Cell. Cardiol.

[b52] Weitzel LB, Ambardekar AV, Brieke A, Cleveland JC, Serkova NJ, Wischmeyer PE (2013). Left ventricular assist device effects on metabolic substrates in the failing heart. PLoS ONE.

[b53] Williams H, King N, Griffiths EJ, Suleiman MS (2001). Glutamate-loading stimulates metabolic flux and improves cell recovery following chemical hypoxia in isolated cardiomyocytes. J. Mol. Cell. Cardiol.

[b54] Wischmeyer PE, Jayakar D, Williams U, Singleton KD, Riehm J, Bacha EA (2003). Single dose of glutamine enhances myocardial tissue metabolism, glutathione content, and improves myocardial function after ischemia–reperfusion injury. JPEN J. Parenter. Enteral Nutr.

[b55] Zachara NE, Hart GW (2006). Cell signaling, the essential role of O-GlcNAc!. Biochim. Biophys. Acta.

